# Research and Application of Chondroitin Sulfate/Dermatan Sulfate-Degrading Enzymes

**DOI:** 10.3389/fcell.2020.560442

**Published:** 2020-12-03

**Authors:** Wenshuang Wang, Liran Shi, Yong Qin, Fuchuan Li

**Affiliations:** National Glycoengineering Research Center and Shandong Provincial Key Laboratory of Carbohydrate Chemistry and Glycobiology, Shandong University, Jinan, China

**Keywords:** chondroitin sulfate, dermatan sulfate, structure-function relationships, oligosaccharide, enzymes

## Abstract

Chondroitin sulfate (CS) and dermatan sulfate (DS) are widely distributed on the cell surface and in the extracellular matrix in the form of proteoglycan, where they participate in various biological processes. The diverse functions of CS/DS can be mainly attributed to their high structural variability. However, their structural complexity creates a big challenge for structural and functional studies of CS/DS. CS/DS-degrading enzymes with different specific activities are irreplaceable tools that could be used to solve this problem. Depending on the site of action, CS/DS-degrading enzymes can be classified as glycosidic bond-cleaving enzymes and sulfatases from animals and microorganisms. As discussed in this review, a few of the identified enzymes, particularly those from bacteria, have wildly applied to the basic studies and applications of CS/DS, such as disaccharide composition analysis, the preparation of bioactive oligosaccharides, oligosaccharide sequencing, and potential medical application, but these do not fulfill all of the needs in terms of the structural complexity of CS/DS.

## Structure and Functions of Chondroitin Sulfate/Dermatan Sulfate

**FIGURE 1:**
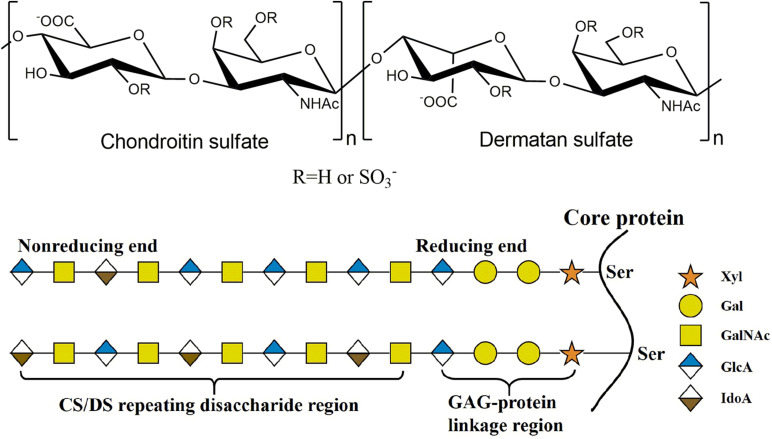
The structure of CS/DS. The CS/DS chain consisting of D-glucuronic acid (GlcA) or L-iduronic (IdoA) acid glycosidically linked to *N*-acetylgalacyosamine (GalNAc) [(-4GlcAβ1-3GalNAcβ1-) or (-4IdoAα1-3GalNAcβ1-). CS/DS chains are covalently attached to the core protein by GAG-protein linkage region tetrasaccharide.

As a major member of the glycosaminoglycan (GAG) family, chondroitin sulfate (CS)/dermatan sulfate (DS) chains covalently attach to core proteins to form CS/DS proteoglycans (CS/DSPGs), which are widespread on cell surfaces and within extra/pericellular matrices to regulate the extracellular environment, involving in many biological and pathophysiological activities (Sugahara and Kitagawa, 2000). As the side chains of PGs, CS is composed of repeating disaccharides consisting of D-glucuronic acid (GlcA) and *N*-acetylgalactosamine (GalNAc) with different sulfation patterns, once the GlcA residues are isomerized to L-iduronic acid (IdoA) residues CS is converted to DS, also called CS-B, and CS and DS domains are usually detected in one chain as co-hybrid structure CS/DS (Sugahara et al., 2003; Figure 1). The sulfated modification of CS/DS chains at C-4 and/or C-6 of GalNAc or/and C-2 of GlcUA/IdoUA by various specific sulfotransferases generates significant structural diversity (Kusche-Gullberg and Kjellen, 2003; Figure 1). Monosulfated disaccharide GlcAβ1-3GalNAc(4S) (A unit) and GlcA β1-3GalNAc(6S) (C unit), of which 4S and 6S stand for 4-*O*-sulfate and 6-*O*-sulfate, respectively, are the most common components found in CS from terrestrial animals (Mathews, 1958; Table 1). Additionally, some highly sulfated disaccharides, such as GlcA(2S)β1-3GalNAc(6S) (D unit), of which 2S stands for 2-*O*-sulfate, and GlcAβ1-3GalNAc(4S, 6S) (E unit), have been found in CS/DS from mammals, in which they account a relatively low proportion but play very important roles in various functions of CS/DS chains (Nandini and Sugahara, 2006; Table 1). In contrast, DS from mammals is mainly composed of the iA unit (IdoAα1-3GalNAc(4S)) with a small amount of the iB unit (IdoA(2S)α1-3GalNAc(4S)) (Bao et al., 2005; Nandini and Sugahara, 2006). Interestingly, some CS/DS chains from marine animals contain a high proportion of rare highly sulfated disaccharides, such as the D unit in CS from shark fin and the E unit in CS from squid cartilage (Mizumoto et al., 2013a; Ueoka et al., 2000; Table 1). Traditionally, CS/DS is named based the main common disaccharide unit or enriched rare disaccharide unit, such as CS-A from mammalian cartilage and sturgeon notochord containing A unit as main disaccharide, CS-C from shark cartilage containing C unit as main disaccharide, CS-D from shark fin containing rare D unit, and CS-E from squid cartilage containing rare E unit. CS/DS chains are widely present in connective tissues of vertebrates and invertebrates, and the polymerization degree and sulfation pattern of CS/DS polysaccharide chains determine the physicochemical properties and physiological and pharmacological activities of CS/DS and CS/DSPGs. The structure complexity of CS/DS leads to its functional diversity. More and more studies have shown that CS/DS is involved in cell division and differentiation (Sugahara et al., 2003; Mizuguchi et al., 2003; Bülow and Hobert, 2006; Izumikawa et al., 2010; Schwartz and Domowicz, 2018; Shida et al., 2019), cell adhesion (Sugahara et al., 2003; Handel et al., 2005; Bülow and Hobert, 2006; Sugahara and Mikami, 2007), morphogenesis (Klüppel et al., 2005; Hayes et al., 2018), inflammation (Li et al., 2020; Campo et al., 2009) and viral infection (Hsiao et al., 1999; Bergefall et al., 2005; Kim et al., 2017). CS/DS chains carry out these functions through interacting with target proteins such as various growth factors fibroblast growth factor (FGF), hepatocyte growth factor (HGF) and pleiotrophin (PTN) (Nandi et al., 2006; Taylor and Gallo, 2006) and cytokines (Hwang et al., 2003; Mizuguchi et al., 2003; Izumikawa et al., 2004; Mizumoto and Sugahara, 2013).

**TABLE 1:** The CS/DS disaccharide and unsaturated disaccharide produced by CS/DS lyase.

CS units		DS units		Unsaturated units	

Unit	Sequence	Unit	Sequence	Unit	Sequence
**O unit**	GlcAβ1-3GalNAc	**iO unit**	IdoAα1-3GalNAc	**ΔO unit**	ΔHexA-GalNAc
**A unit**	GlcAβ1-3GalNAc(4S)	**iA unit**	IdoAα1-3GalNAc(4S)	**ΔA unit**	ΔHexA-GalNAc(4S)
**B unit**	GlcA(2S)β1-3GalNAc(4S)	**iB unit**	IdoA(2S)α1-3GalNAc(4S)	**ΔB unit**	ΔHexA(2S)-GalNAc(4S)
**C unit**	GlcAβ1-3GalNAc(6S)	**iC unit**	IdoAα1-3GalNAc(6S)	**ΔC unit**	ΔHexA-GalNAc(6S)
**D unit**	GlcA(2S)β1-3GalNAc(6S)	**iD unit**	IdoA(2S)α1-3GalNAc(6S)	**ΔD unit**	ΔHexA(2S)-GalNAc(6S)
**E unit**	GlcAβ1-3GalNAc(4S,6S)	**iE(H) unit**	IdoAα1-3GalNAc(4S,6S)	**ΔE unit**	ΔHexA-GalNAc(4S,6S)
**F unit**	GlcAβ1-3GalNAc(4S) — Fuc(α1-3)	**iK unit**	IdoA(3S)α1-3GalNAc(4S)	**ΔF unit**	ΔHexA-GalNAc(4S) — Fuc(α1-3)
**G unit**	GlcAβ1-3GalNAc(4S) — Glc(β1-6)	**iL unit**	IdoA(3S)α1-3GalNAc(6S)	**ΔG unit**	ΔHexA-GalNAc(4S) — Glc(β1-6)
**K unit**	GlcA(3S)β1-3GalNAc(4S)	**iT unit**	IdoA(2S)α1-3GalNAc(4S,6S)	ΔK unit*	ΔHexA(3S)-GalNAc(4S)
**L unit**	GlcA(3S)β1-3GalNAc(6S)	**iU unit**	IdoA(2S)α1-3GalNAc	ΔL unit*	ΔHexA(3S)-GalNAc(6S)
**M unit**	GlcA(3S)β1-3GalNAc(4S,6S)			ΔM unit*	ΔHexA(3S)-GalNAc(4S,6S)
**T unit**	GlcA(2S)β1-3GalNAc(4S,6S)			**ΔT unit**	ΔHexA(2S)-GalNAc(4S,6S)
**U unit**	GlcA(2S)β1-3GalNAc			**ΔU unit**	ΔHexA(2S)-GalNAc

*The disaccharides containing ΔHexA (3S) are unstable and result in sulfated GalNAc.

## CS/DS Biosynthesis

The biosynthesis of CS/DS is a complex, multistep and enzymatically accommodated process that occurs in endoplasmic reticulum/Golgi compartments and is initiated by the synthesis of GAG-protein linkage region covalently linked to specific serine residues embedded in different core proteins (Sugahara and Kitagawa, 2000; Silbert and Sugumaran, 2002; Sugahara et al., 2003). The linkage region is a specific tetrasaccharide structure GlcAβ1-3Galβ1-3Galβ1-4Xylβ1, in which Gal and Xyl represent galactose and xylose residues, respectively (Figure 1). This structure is catalyzed by the corresponding glycosyltransferase in the tetrasaccharide sequence. Firstly, a Xyl residue is connected to a specific Ser residue of core protein through the catalyzation of xylosyltransferase (Götting et al., 2000, 2007); then, β1,4-galactosyltransferase I (Almeida et al., 1999; Okajima et al., 1999) and β1,3-galactosyltransferase II (Bai et al., 2001) catalyze the connection of two Gal residues in turn; and, finally, β1,3-glucuronyltransferase I (Kitagawa et al., 1998; Bai et al., 1999, 2001) catalyzes the addition of GlcUA residue to form the tetrasaccharide linkage region.

Once the synthesis of the linkage tetrasaccharide is completed, the extension of CS/DS chain will be triggered by the transfer of a GalNAc residue to the nonreducing terminal GlcA residue of the tetrasaccharide linkage region by GalNAc transferase I, and then GlcA and GalNAc residues will be added in turn to form the chondroitin (Chn) skeleton composed of repeating disaccharide GlcA-GlaNAc through alternating catalysis of GalNAc transferase II and GlcA transferase II (Sugahara and Kitagawa, 2000; Silbert and Sugumaran, 2002; Sugahara et al., 2003). During the process of polymerization, some GlcA residues in the Chn skeleton can be transformed into IdoA under the control of two GlcA C-5 epimerases, thereby transforming the corresponding Chn domains into its stereoisomer dermatan domains (Maccarana et al., 2006; Pacheco et al., 2009). Furthermore, some hydroxyl groups of GalNAc or GlcA/IdoA residues in the chains can be site-specifically modified by a variety of sulfotransferases by using 3’-phosphoadenosine 5’-phosphosulfate as a donor substrate (Habuchi, 2000). Under the control of chondroitin 4-*O*-sulfotransferase-1, 2 and 3 (Yamauchi et al., 2000; Hiraoka et al., 2000; Kang et al., 2002), and dermatan 4-*O*-sulfotransferase (Evers et al., 2001; Mikami et al., 2003) the sulfate group is transferred to the hydroxyl group at the C-4 location of GlcA to generate an A unit and an iA unit, respectively. The 6-*O*-sulfation of the C unit is catalyzed by chondroitin 6-*O*-sulfotransferase-1 (Fukuta et al., 1998). The GalNAc 4-sulfate 6-*O*-sulfotransferase transfers sulfate to the C-6 position of the A/iA unit to generate an E/iE unit (Ohtake et al., 2001), and uronyl 2-*O*-sulfotransferase sulfates GlcA in the C-2 position of the C/iA unit to generate a D/iB unit (Kobayashi et al., 1999). The space-time-dependent expression and combined action of these enzymes make the structure of CS/DS chains extremely diverse and complex, which presents significant challenges for the structural and functional studies of CS/DS.

## CS/DS-Degrading Enzymes

As a reverse process of CS/DS synthesis, the degradation of CS/DS chains in the organisms also involves various enzymes including glycosidic bond-cleaving enzymes and sulfatases, which correspond to glycosyltransferases and sulfotransferases, respectively. Thus, the CS/DS-degrading enzymes are indispensable tools for analyzing the structure and function of CS/DS chains. Based on the enzymatic mechanism, CS/DS glycosidic bond-cleaving enzymes are accordingly classified as either hydrolases or lyases. According to their similarities of amino acid sequences, hydrolases and lyases are assigned to glycoside hydrolase (GH) families and polysaccharide lyase (PL) families, respectively (Henrissat, 1991). CS/DS hydrolases and lyases are usually found in animals and microorganisms, respectively. CS/DS sulfatases belong to the formylglycine-dependent family that specifically hydrolyzes sulfate esters on poly- and oligosaccharides of CS/DS. These CS/DS-degrading enzymes play key roles in the catabolic metabolism of CS/DS polysaccharides and are widely found in animals and microorganisms.

### Hydrolases

In animals, CS/DS glycosidic bond-cleaving enzymes are hydrolases that cleave the β-1,4-glycosidic bond between GalNAc and GlcA residues in CS chains via a hydrolysis mechanism to produce saturated oligosaccharide products (Kreil, 1995). In mammals, the so-called hyaluronidases (EC 3.2.1.35) have been reported to be the only hydrolases that cleave CS chains, and even some hyaluronidases do not degrade hyaluronan (HA) but only degrade CS (Kaneiwa et al., 2010). In fact, most animal-derived hyaluronidases and microorganism-derived CS/DS lyases, which we will introduce later, show both HA- and Chn/CS-degrading activities, which may be due to the very similar structural features of the two GAG polysaccharides. Both HA and CS chains have same types of β-glycosidic bonds in and between repeating disaccharide units consisting of GlcA and hexosamine residues, and the only difference in structure between them is that the acetylated hexosamine GlcNAc in HA is replaced by the GalNAc in the Chn skeleton of CS. In addition, like other glycoside hydrolases, hyaluronidases exhibit certain transglycosidase activities (Hoffman et al., 1956), which can be used for synthesizing of HA (Kobayashi et al., 2003), Chn (Kobayashi et al., 2003), CS (Fujikawa et al., 2005), their derivatives (Ochiai et al., 2007b; Kobayashi et al., 2003), and hybrids of HA-Chn and HA-CS (Ochiai et al., 2007a).

**FIGURE 2:**
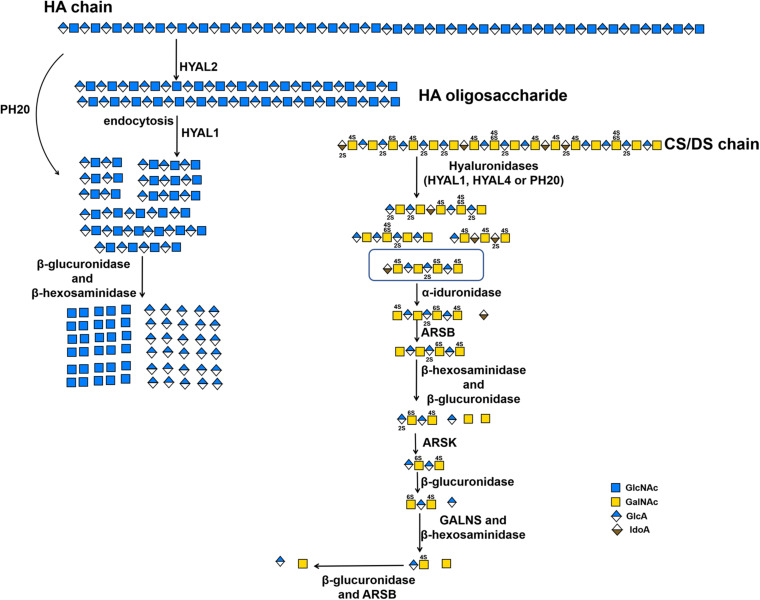
The substrate degradation properties of hyaluronidases and sulfatases in animals. IDS, iduronate-2-sulfatase; ARSK, glucurono-2-sulfatase; GALNS, *N*-acetylgalactosamine-6-sulfatase; ARSB, *N*-acetylgalactosamine-4-sulfatase.

In human genome, six highly homologous genes have been found to encode hyaluronidase-like sequences including five functional hyaluronidase*s* genes (*HYAL1, HYAL2, HYAL3, HYAL4 and SPAM1*) and a pseudogene *HYALP1*(also called *HYAL6P*) that is transcribed in humans but is not translated (Csoka et al., 2001; Stern and Jedrzejas, 2006). *HYAL1, HYAL2*, and *HYAL3* are clustered in the chromosome 3p21.3 locus, whereas the *HYAL4*, *SPAM1*(encodes PH-20) and *HYALP1* genes are found on chromosome 7q31.3 (Csoka et al., 2001; Stern, 2003). Based on the optimal pH, most of these hyaluronidases show their highest activity at an acidic pH (Lokeshwar et al., 2001; Sabeur et al., 1997). As mentioned above, these so-called hyaluronidases from humans also show a certain degree of CS-degrading activity. The sperm-specific enzyme PH-20 shows much higher activity against Chn than CSA and HA at pH 4.5, whereas prefers HA and CS to Chn at pH 4.0 (Honda et al., 2012). In contrast, plasma hyaluronidase HYAL1 prefers to degrade CS-A than HA at pH 4.5 but prefers HA than CS-A at pH 3.5 (Csoka et al., 2001; Honda et al., 2012; Yamada, 2015). Furthermore, C units in CS-C negatively affect the hyaluronidase activity of PH-20. More interestingly, HYAL4 has been shown to be a specific CS-degrading enzyme without any activity toward HA, which means that the name of hyaluronidase is a misnomer for this enzyme (Csoka et al., 2001; Stern, 2003; Jedrzejas and Stern, 2005; Kaneiwa et al., 2010). The HYAL1 and PH-20 with CS-degrading activity cannot cleave the galactosaminidic linkages in -GalNAc-IdoA- and -GalNAc-GlcA(2S)- sequences, which are often found in DS chains and in highly sulfated D unit-containing domains of CS chains, respectively. In contrast, HYAL4 could strongly cleave the galactosaminidic linkages in -GlcA(2S)-GalNAc(6S)-GlcA-GalNAc(4S or 6S)- (Kaneiwa et al., 2010), suggesting that HYAL4 plays an important role in the degradation of CS in mammal. However, the degradation mechanism of DS chains in animals remains to be further investigated. Enzymes HYAL1 and HYAL2 are the main HA-degrading enzymes in somatic tissues. HYAL1 is a lysosomal enzyme and by contrast HYAL2 binds to plasma membrane via a glycosylphosphatidylinositol anchor (Afify et al., 1993; Rai et al., 2001). The traditional HA degradation model is that high molecular weight HA is digested into low molecular weight HA oligosaccharides by extracellular HYAL2, then, the HA oligosaccharides are internalized by interaction with cell surface receptors, and the internalized HA oligosaccharides are further decomposed by lysosomal HYAL1, exoglycosidases β-glucuronidase and β-hexosaminidase (Hex) (Stern, 2003; Stern, 2004; Figure 2 and Table 2). As a dimeric enzyme, human Hex exists in two main isoforms HexA (αβ) and HexB (ββ), of which the α- and β-subunits are encoded by *HexA* and *HexB* genes, respectively (Chiricozzi et al., 2014). Interestingly, HexA can hydrolyze HA and CS chains from their non-reducing ends but HexB cannot (Thompson et al., 1973; Bearpark and Stirling, 1978). Like HA, CS can be internalized by interaction with cell surface receptors and degraded in the lysosome (Wood et al., 1973). Indeed, mice lost both HYAL1 and Hex activity show the accumulation of HA and CS (Gushulak et al., 2012).

**TABLE 2:** CS/DS-degrading enzymes from bacteria.

Name	Substrate	Source	Degradation mechanism	Action pattern	EC number (Filmay)	References
**CSase ABC I**	HA, CS or DS	*Proteus vulgaris*	lysis	endo	EC 4.2.2.20 (PL8)	Yamagata et al., 1968; Hamai et al., 1997
**CSase ABC II**	HA, CS or DS	*Proteus vulgaris*	lysis	exo (from nonreducing end)	EC 4.2.2.20 (PL8)	Yamagata et al., 1968; Hamai et al., 1997
**CSase AC I**	HA or CS	*Flavobacterium heparinum*	lysis	endo	EC 4.2.2.5 (PL8)	Yamagata et al., 1968; Gu et al., 1995
**CSase AC II**	HA or CS	*Arthrobacter aurescens*	lysis	exo (from reducing end)	EC 4.2.2.5 (PL8)	Lunin et al., 2004; Yin et al., 2016
**CSase AC-III**	HA or CS	*Flavobacterium* sp. Hpl02	lysis	endo	EC 4.2.2.5 (PL8)	Miyazono et al., 1990
**CSase C**	HA or CSC	*Flavobacterium heparinum.*	lysis	endo	EC 4.2.2.-(PL8)	Michelacci and Dietrich, 1976
**CSase B**	DS	*Flavobacterium heparinum*	lysis	endo	EC 4.2.2.19 (PL6)	Yamagata et al., 1968; Gu et al., 1995
**CSase B II**	DS	*Flavobacterium* sp. Hpl02	lysis	endo	EC 4.2.2.19 (PL6)	Miyazono et al., 1990
**Hyaluronidase-B**	HA or CS	*Bacillus* sp. A50	lysis	endo	EC 4.2.2.- (PL8)	Guo et al., 2014
**AcODV-E66**	HA or CS	*Autographa californica* nucleopolyhedrovirus	lysis	endo	EC 4.2.2.- (PL8)	Sugiura et al., 2011
**BmODV-E66**	HA or CS	*Bombyx mori nucleopolyhedrovirus*	lysis	endo	EC 4.2.2.- (PL8)	Sugiura et al., 2013
**HCLase**	HA or CS	*Vibrio* sp. FC509	lysis	endo	EC 4.2.2.- (PL8)	Han et al., 2014
**HCDLase**	HA, CS or DS	*Vibrio* sp. FC509	lysis	exo (from reducing end)	EC 4.2.2.- (PL8)	Wang et al., 2017
**HCLase Er**	HA or CS	*Vibrio* sp. FC509	lysis	endo	EC 4.2.2.- (PL8)	(Peng et al., 2018)
**BniHL**	HA or CS	*Bacillus niacin*	lysis	endo	EC 4.2.2.- (PL8)	Kurata et al., 2015
**ChoA1**	HA or CS	*Arthrobacter* sp. MAT3885	lysis	endo	EC 4.2.2.- (PL8)	Kale et al., 2015
**BtCDH**	HA or CS	*Bacteroides thetaiotaomicron*	lysis	endo	EC 4.2.2.-(PL8)	Ndeh et al., 2018
**BHCSase AC**	HA or CS	*Helicobacter bizzozeronii*	lysis	endo	EC 4.2.2.-(PL8)	Namburi et al., 2016
**AsChnAC**	HA or CS	*Arthrobacter* sp. SD-04	lysis	exo (undetermined)	EC 4.2.2.- (PL8)	Chen et al., 2019
**HYAL**	HA	*Streptomyces hyalurolyticus*	lysis	endo	EC 4.2.2.1 (PL8)	Ohya and Kaneko, 1970
**HYAL**	HA	*Streptococcus dysgalactiae*	lysis	endo	EC 4.2.2.1 (PL8)	Sting et al., 1990
**4-*O*-endosulfatase**	4-*O*-sulfate on GalNAc of CS and DS	*Vibrio* sp. FC509	sulfatase	endo (from reducing end)	EC 3.1.6.- (S1_27)	Wang et al., 2015; Wang et al., 2019b
**BT_3349**	4-*O*-sulfate on GalNAc of CS and DS	*Bacteroides thetaiotaomicron*	sulfatase	endo	EC 3.1.6.- (S1_27)	Ulmer et al., 2014
**BT_3333**	6-*O*-sulfate on GalNAc	*Bacteroides thetaiotaomicron*	sulfatase	exo (nonreducing end)	EC 3.1.6.- (S1_15)	Ulmer et al., 2014
**BT_1596**	2-*O*-sulfate on ΔHexA of HS/CS degradation products	*Bacteroides thetaiotaomicron*	sulfatase	exo (nonreducing end)	EC 3.1.6.- (S1_9)	Ulmer et al., 2014
**chondro-4-sulfatase**	4-*O*-sulfate on GalNAc of CS	*Proteus vulgaris*	sulfatase	exo (reducing end)	EC 3.1.6.9 (S1_27)	Yamagata et al., 1968; Sugahara and Kojima, 1996
**chondro-6-sulfatase**	6-*O*-sulfate on GalNAc of CS	*Proteus vulgaris*	sulfatase	exo (reducing end)	EC 3.1.6.10 (S1_15)	Yamagata et al., 1968; Sugahara and Kojima, 1996
**delta-hexuronate-2-sulfatase**	2-*O*-sulfate on ΔHexA of HS/CS degradation products	*Flavobacterium heparinum*	sulfatase	exo (nonreducing end)	EC 3.1.6.- (S1_9)	Sugahara and Kojima, 1996; Myette et al., 2003
**PB2SF**	2-*O*-sulfate on ΔHexA of HS/CS degradation products	*Photobacterium* sp. FC615	sulfatase	exo (reducing end)	EC 3.1.6.- (S1_2)	Wang et al., 2019a
**exoPB4SF**	4-*O*-sulfate on CS/DS GalNAc	*Photobacterium* sp. FC615	sulfatase	exo (reducing end)	EC 3.1.6.12 (S1_27)	Wang et al., 2019b

*PL: Polysaccharide lyase family; S: Sulfatase famlily.*

### Lyases

Unlike the hyaluronidases from animals, CS/DS-lyases from microorganisms cleave the β-1,4-glycosidic linkage between hexosamine and hexuronic acid residues in HA or CS/DS chains through an elimination reaction to yield an unsaturated double bond between C-4 and C-5 on the uronic acid residue Δ^4,5^hexuronate (ΔHexA) at the nonreducing end of the resulting oligosaccharide products. Conventionally, CD/DS lyases are named as chondroitinases (CSases), although some of them cannot digest CS, such as CSase B, which shows specific activity towards DS only. Moreover, CS/DS lyases can be classified into endolytic and exolytic types according to their substrate-degrading patterns, in which endolyases cleave CS/DS chains initially into larger oligosaccharides and finally to small disaccharides with a random cleavage pattern, whereas exolyases successively release disaccharides from the end of the sugar chains and do not produce any larger oligosaccharides in the process. The unsaturated double bond of oligosaccharides produced by lyases shows specific absorption of ultraviolet light at 232 nm, which is beneficial for the detection of CS/DS oligosaccharide products. In addition, CS/DS lyases have many advantages such as more diversity, better stability and activity and simpler preparation compared with hydrolases. Due to the outstanding features above, lyase has a wide range of applications in the preparation of oligosaccharides (Li et al., 2007; Mizumoto et al., 2013a), treatment of nerve damage (Janzadeh et al., 2017; Mondello et al., 2015; Sarveazad et al., 2017), and other CS structure-activity relationship studies.

#### Commercialized CS/DS Lyases

**FIGURE 3:**
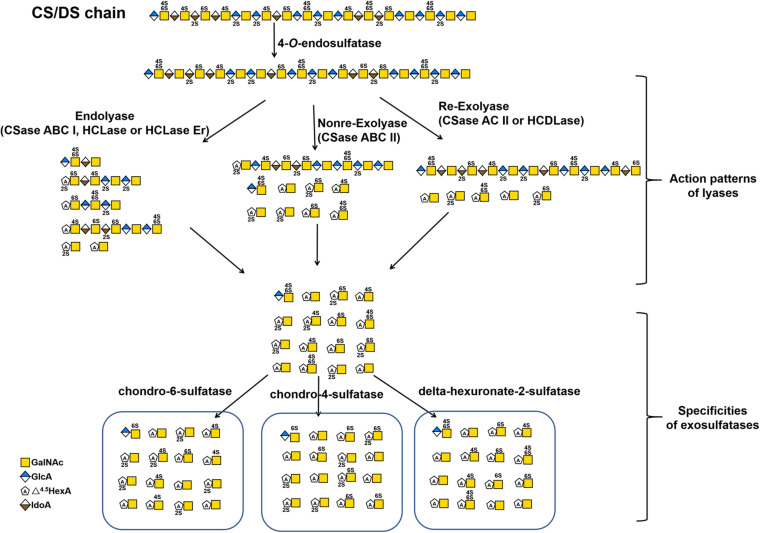
The substrate degradation properties of lyases and sulfatases in bacteria. Nonre-Exolyase, exolyase acted on the nonreducing end of CS/DS chains; Re-Exolyase, exolyase acted on the reducing end of CS/DS chains.

Based on their substrate specificity, CS/DS lyases are subdivided into three types CSase ABC, AC and B. The CSase ABC can digest CS, DS and HA, irrespective of their sulfation/C-5-epimerization pattern. Currently, the CSase ABC from *Proteus vulgaris* is widely used for GAG structure analysis. The commercially available CSase ABC comprises a mixture of the CSase ABC I (EC 4.2.2.20) with endolytic activity and the CSase ABC II (EC 4.2.2.21) with exolytic activity (Yamagata et al., 1968; Hamai et al., 1997). The CSase AC (EC 4.2.2.5) is highly sensitive to the 5-epimerization of GlcA residues in GAG chains and can act only on CS, HA and CS domains in CS-DS hybrid chains (Yamagata et al., 1968; Hiyama and Okada, 1975; Linhardt et al., 2006), whereas, the CSase B (EC 4.2.2.19) is specifically cleaves DS and DS domains in CS-DS hybrid chains (Yamagata et al., 1968; Gu et al., 1995). The CSase AC I from *Flavobacterium heparinum* and CSase AC II from *Arthrobacter aurescens* are well-known CS/DS lyases showing endolytic and exolytic activities, respectively (Yamagata et al., 1968; Hiyama and Okada, 1975). The CSase B from *Flavobacterium heparinum* is the only commercialized lyase with specific endolytic activity to DS (Yamagata et al., 1968). Notably, the CSase ABC and CSase AC belong to the polysaccharide lyase (PL) family 8, but the CSase B belongs to PL family 6, which comprises alginate lyases (www.cazy.org) (Figure 3 and Table 2). Structural heterogeneity has hampered structure-function relationship studies of CS/DS chains. However, there are only a few CS/DS lyases that have been characterized in detail and commercially available, which is far from meeting the needs of CS/DS-related researches and applications.

#### Newly Identified CS/DS Lyases

Most recently, some unique CS/DS lyases have been identified in various microorganisms (Table 2). Most of them are CS and HA lyases. Hyaluronidase-B from *Bacillus* sp. A50 and the hyaluronate lyase BniHL from the deep-sea bacterium *Bacillus niacin* show endolytic activity towards CS and HA at an approximately neutral pH (Guo et al., 2014; Kurata et al., 2015). A Chn lyase ChoA1 from *Arthrobacter* sp. MAT3885 can degrade Chn, CS and HA (Kale et al., 2015). Namburi et al. found a CSase AC from *Helicobacter bizzozeronii* in canine stomach, and this enzyme exhibited specific endolytic activity on HA and various CSs with different sulfation patterns at an optimum low pH between pH 4.0 and pH 5.5 and might represent one of several factors involved in the development of gastritis in dogs (Namburi et al., 2016). The CSase AsChnAC identified from *Arthrobacter* sp. SD-04 displays exolytic activity toward HA and various CSs too (Chen et al., 2019). Sugiura et al. identified two highly homologous occlusion-derived variants of virus envelope protein 66s from *Autographa californica* nucleopolyhedrovirus and *Bombyx mori* nucleopolyhedrovirus, and both of the variants showed specific lyase activity towards non-sulfated and 6-*O*-sulfated CS (Sugiura et al., 2011; Sugiura et al., 2013). BtCDH from the human gut microbe *Bacteroides thetaiotaomicron* belongs to a new PL family (PL29) and shows optimum endolytic activity toward HA and CS, particularly large chains longer than decasaccharide, at a very high temperature of 60 °C (Ndeh et al., 2018). Two novel CS/DS lyases have been found from *Acinetobacter* sp. C26 and *Sphingomonas paucimobilis*, respectively, both of which have lower molecular weights but similar broad-spectrum activities against CS, DS and HA compared with CSase ABC (Zhu et al., 2017; Fu et al., 2018). However, most of these studies have mainly focused on the basic enzymatic properties and rough substrate specificity of these novel CS/DS lyases, and there is a lack of in-depth studies on their substrate-degrading mode and catalytic mechanism of these enzymes.

Marine animals are enriched in CS/DS with unique structures, which indicates that there must be a large number of microorganisms owning corresponding enzymes that would allow them to degrade and utilize these unique CS/DS forms in the ocean. Consistent with our speculation, marine bacteria-derived CS/DS-degrading enzymes show various unique characteristics. HCLase is the first marine-derived CS/DS lyase identified from the bacterium *Vibrio* sp., which is isolated from sea mud. This enzyme has high endolytic activity towards HA and CS with various sulfation patterns at an approximately neutral pH and exhibits excellent biochemical characteristics, such as halophilicity, pH stability and thermal stability. Although HCLase can digest the β-1,4-glycosidic bond between GalNAc and most disaccharide units, it is unable to act on the galactosaminidic linkage between GalNAc and the D unit, suggesting that the 2-*O*-sulfation of GlcA inhibits the action of HCLase, which is similar to the case of CSase AC I (Han et al., 2014). In contrast, HCLase Er from the same strain is the first identified CS lyase that is specifically inhibited by both 4-*O*- and 6-*O*-sulfation of GalNAc, which is very useful for selectively preparing E unit–rich oligosaccharides from CS polysaccharides (Peng et al., 2018). HCDLase is a novel exo-type lyase from the same bacterial strain, which can degrade HA, CS and DS from their reducing end by sequentially releasing unsaturated disaccharides. In particular, it can effectively cleave CS oligosaccharides with reducing ends that are labeled with 2-aminobenzamide (2-AB) to release the 2-AB-labelled reducing-end disaccharides, which is a rare activity useful for the enzymatic sequencing of CS chain (Wang et al., 2017). Taken together, these studies suggest that the ocean is an untapped treasure trove of new CS/DS-degrading enzymes.

### CS/DS Sulfatases

The sulfation patterns of CS/DS chains play a key role in various functions of CS/DS. Technically, sulfatases with specific activity that allow them to selectively remove sulfate groups from CS/DS chains should be another important tool for the structural and functional studies of CS/DS. Based on the positions of sulfate groups in CS/DS chains, there are several types of specific sulfatases in animals and bacteria, such as *N*-acetylgalactosamine-4-*O*-sulfatase (Yamagata et al., 1968; Sugahara and Kojima, 1996; Wang et al., 2019b; Baum et al., 1959) and *N*-acetylgalactosamine-6-*O*-sulfatase (Yamagata et al., 1968; Sugahara and Kojima, 1996; Lim and Horwitz, 1981; Singh et al., 1976), which specifically hydrolyze sulfate groups on the C-4 and C-6 positions of GalNAc residues, respectively, and hexuronate-2-*O*-sulfatase, which specifically removes C-2 sulfate groups from saturated or unsaturated hexuronic acids derived from the digestion of CS/DS by GAG lyases (Sugahara and Kojima, 1996; Myette et al., 2003; Wang et al., 2019a; Tables 2, 3). Based on the sequence similarities, the GAG sulfatases were recently classified in the database SulfAtlas (http://abims.sb-roscoff.fr/sulfatlas/) (Barbeyron et al., 2016). Animal CS/DS sulfatases are lysosomal enzymes responsible for the degradation of endogenous CS/DS, and genetic defects of these enzymes result in the formation of several mucopolysaccharidoses (MPS) in humans, such as MPS II, MPS IVA and MPS VI (Khan et al., 2017). The well-studied animal CS/DS sulfatases are *N*-acetylgalactosamine-4-*O* sulfatase (also named Arylsulfatase B, ARSB) (Baum et al., 1959; Wicker et al., 1991), *N*-acetylgalactosamine-6-*O*-sulfatase (GALNS) (Lim and Horwitz, 1981; Bielicki and Hopwood, 1991; Tomatsu et al., 1991), iduronate-2-*O*-sulfatase (IDS) (Lim et al., 1974; Shaklee et al., 1985; Wilson et al., 1990) and Glucurono-2-sulfatase (Arylsulfatase K, ARSK) (Dhamale et al., 2017), which specifically remove the 4-*O*-sulfate group from sulfated GalNAc residues of CS/DS, 6-*O*-sulfate group from sulfated GalNAc residues of CS/DS and sulfated galactose of keratan sulfate, 2-*O*-sulfate groups of sulfate IdoA residues of DS and heparin (Hep), and 2-*O*-sulfate groups of GlcA residues of heparan sulfate (HS), respectively (Parenti et al., 1997; Tables 2, 3). All these animal CS/DS sulfatases belong to exosulfatases.

**TABLE 3:** CS/DS-degrading enzymes from animals.

Name	Substrate	Source	Degradation mechanism	Action pattern	EC number	References
**HYAL1**	HA or CS	*human*	hydrolysis	endo	EC 3.2.1.35 (GH56)	De Salegui and Pigman, 1967; Csoka et al., 1999
**HYAL2**	HA	*human*	hydrolysis	endo	EC 3.2.1.35 (GH56)	Rai et al., 2001; Csoka et al., 1999
**HYAL3**	HA	*human*	hydrolysis	endo	EC 3.2.1.35 (GH56)	Csoka et al., 1999
**HYAL4**	CS	*human*	hydrolysis	endo	EC 3.2.1.35 (GH56)	Csoka et al., 1999; Kaneiwa et al., 2010
**PH-20**	HA or CS	*human*	hydrolysis	endo	EC 3.2.1.35 (GH56)	Cherr et al., 2001
**Hyaluronidase**	HA	*bovine* testis	hydrolysis	endo	EC 3.2.1.35 (GH56)	Freeman et al., 1949
**HYAL**	HA	*bee venom*	hydrolysis	endo	EC 3.2.1.35 (GH56)	Gmach and Kreil, 1993
***N*-acetylgalactosamine-4-sulfatase (Arylsulfatase B, ARSB)**	4-*O*-sulfate on GalNAc of CS and DS	*human*	sulfatase	exo (reducing end)	EC 3.1.6.12 (S1_2)	Baum et al., 1959; Wicker et al., 1991
***N*-acetylgalactosamine-6-sulfatase (GALNS)**	6-*O*-sulfate on GalNAc of CS and keratan sulfate	*human*	sulfatase	exo (reducing end)	EC 3.1.6.4 (S1_5)	Lim and Horwitz, 1981; Bielicki and Hopwood, 1991; Tomatsu et al., 1991
**Iduronate-2-sulfatase (IDS)**	2-*O*-sulfate on IdoA of DS and Hep	*human*	sulfatase	exo (nonreducing end)	EC 3.1.6.13 (S1_7)	Lim et al., 1974; Shaklee et al., 1985; Wilson et al., 1990
**Glucurono-2-sulfatase (Arylsulfatase K, ARSK)**	2-*O*-sulfate on GlcA of HS	*human*	sulfatase	exo (nonreducing end)	EC 3.1.6.18 (S1_7)	Dhamale et al., 2017

*GH: Glycoside hydrolase family, S: Sulfatase famlily.*

In contrast, bacterial CS/DS sulfatases are essential for the biodegradation and utilization of CS/DS from animals, and a large number of potential sulfatase genes have been found in the genomes of various bacteria. However, only a few CS/DS sulfatases have been studied in detail. Three Δ^4,5^HexA-2-*O*-sulfatases have been identified from *Flavobacterium heparinum*, *Bacteroides thetaiotaomicron* and *Photobacterium* sp. FC615, that can specifically remove 2-*O*-sulfate ester from a ΔHexA residue located at the nonreducing terminus of an unsaturated oligosaccharide (Sugahara and Kojima, 1996; Myette et al., 2003; Ulmer et al., 2014; Wang et al., 2019a). Two *N*-acetylgalactosamine-4-*O*-sulfatases from *Proteus vulgaris* and *Photobacterium* sp. FC615 specifically hydrolyze 4-*O*-sulfate groups on GalNAc residues at the reducing ends of CS/DS chains (Sugahara and Kojima, 1996; Wang et al., 2019b). An *N*-acetylgalactosamine-6-*O*-sulfatase from *Proteus vulgaris* has been shown to specifically act on 6-*O*-sulfates on GalNAc residues at the reducing termini of CS/DS oligosaccharides (Sugahara and Kojima, 1996), and another *N*-acetylgalactosamine-6-*O*-sulfatase from *Bacteroides thetaiotaomicron* can only attack the 6-*O*-sulfate group on monosaccharide GalNAc residues (Ulmer et al., 2014). Notably, most of the identified CS/DS sulfatases are exo-type enzymes, which only remove sulfate esters from the ends of CS/DS poly-/oligosaccharides and thus have very limited applications to structural and functional studies of CS/DS. Recently, two endo-type *N*-acetylgalactosamine-4-*O*-sulfatases were identified from *Bacteroides thetaiotaomicron* (Ulmer et al., 2014) and *Vibrio* sp. FC509 (Wang et al., 2015), which can effectively remove 4-*O*-sulfate groups from GalNAc residues within the CS/DS chains (Table 2 and Figures 2, 3). Compared with exosulfatases, endosulfatases should be more useful enzymatic tools for CS/DS studies but seem to be very rare in nature. Our recent study has shown that CS/DS sulfatases with similar specificities have common signature sequences and can cluster to form a single branch in the phylogenetic tree, although they descended from separate ancestral genes (Wang et al., 2019a). Based on this finding, a series of Δ^4,5^HexA-2-*O*-sulfatases have been successfully predicted and verified from sequences in GenBank (Wang et al., 2019a), and we believe that some CS/DS endosulfatases could also be found by using this method. In fact, the existence of endosulfatases facilitates the catabolic metabolism of CS/DS by bacteria, in which the degradation of CS/DS chains by lyases can be significantly promoted via pre-desulfation by endosulfatases (Wang et al., 2015).

## Applications in CS/DS Structure-Function Studies

Their great structural heterogeneity endows CS/DS chains with various functions but also poses a great challenge to the structure-function studies of CS/DS. A growing body of research shows that CS/DS chains function through interacting with target proteins and that some oligosaccharide domains with specific structural features in CS/DS chains are involved in these interactions (Sugahara et al., 2003; Raman et al., 2005; Kastana et al., 2019; Pudelko et al., 2019). Thus, it is key for studying the structure and function of CS/DS to investigate the structural features in particular functional domains of the CS/DS chains involved in an interaction with a specific target protein. CS/DS-degrading enzymes with different specific activities play irreplaceable tools in such studies (Linhardt et al., 2006; Li et al., 2010; Wang et al., 2017).

### Compositional Analysis of CS/DS

As a kind of highly heterogeneous polysaccharides, the exact structures of all the chains in CS/DS samples cannot be determined with current technology. Thus, the disaccharide composition is used as a basic parameter to characterize various CS/DS preparations used in basic studies and industrial production. Commercial CS/DS preparations are extracted from terrestrial and marine sources, such as the cartilages from bovine, porcine, chicken, shark and squid, and are wildly used in medicines and health products (Bao et al., 2005; Deepa et al., 2007a; Volpi, 2007, 2009; Valcarcel et al., 2017). However, the biological and pharmacological properties of these CS/DS preparations are seriously affected by the raw materials, manufacturing processes and many other factors impacting their production. Disaccharide analysis has been commonly used to evaluate the quality of CS/DS products. CS/DS lyases play a key role in disaccharide composition assays of CS/DS. In general, the disaccharide compositions of various forms of CS/DS with different sulfation patterns can be easily determined by digestion with the commercial CSase ABC followed by anion-exchange HPLC. However, digestion by CSase ABC causes the conversion of both GlcA and IdoA residues into unsaturated uronic acid and thus CS and DS in the test sample cannot be distinguished by this method. In this case, we can use the substrate specificity of the CSase AC and CSase B to investigate the disaccharide composition and proportions of CS and DS in samples.

For example, to determine the disaccharide composition of CS/DS extracted from shark liver, we used CSases that differed in their specificity (CSase ABC, mixture of CSase AC-I and CSase AC-II or CSase B) to digest the sample, and then the digests were labeled with 2-AB and analyzed by anion exchange HPLC on an amine-bound silica PA-03 column using a solvent system of 16 and 530 mM NaH_2_PO_4_ over a period of 1 h by fluorescent detection. Although the shark liver-derived CS/DS preparation contained highly heterogenous hybrid chains of CS-DS, the disaccharide composition and contents of CS and DS domains in the hybrid chains could be well determined by this method (Li et al., 2007). In summary, CSases with different substrate specificities play an important role in the disaccharide composition assays of CS/DS.

### Preparation of Oligosaccharides With Specific Activity

The various biological functions of CS/DS are thought to be due to their functional domains, some oligosaccharide sequences with specific structural features. For a specific target protein, the functional domains of CS/DS chains are usually not a single specific structure but some oligosaccharide domains with similar characteristics, such as a minimum size requirement and the enrichment of specific oversulfated disaccharide units (Trowbridge and Gallo, 2002; Trowbridge et al., 2002; Sugahara et al., 2003; Sugahara and Mikami, 2007; Li et al., 2010). Therefore, isolation of the functional oligosaccharide domains from CS/DS chains is key to not only structure-function relationship studies of CS/DS but also the preparation of functional oligosaccharides with specific activities. Compared with the harsh conditions of chemical and physical methods, the enzymatic method is mild and biocompatible for degrading CS/DS to prepare the functional domains. In general, functional oligosaccharides with specific activity can be obtained through the partial digestion of CS/DS chains with specific enzymes followed by a series of chromatographic separations, especially affinity chromatography. Various CS/DS glycosidic bond-cleaving enzymes, including hyaluronidases and lyases, have been used to prepare CS/DS oligosaccharides. As mentioned above, the CS/DS oligosaccharides produced by lyases bear an unsaturated 4,5-bond between C-4 and C-5 of ΔHexA at their nonreducing ends, which can be easily detected at 232 nm. Moreover, CS/DS lyases show more flexibility in terms of substrate specificity. Thus, CS/DS lyases, including CSase ABC, AC I and B, have been widely used to partially digest various forms of CS/DS for preparing functional oligosaccharides that specifically bind to certain proteins (Fukui et al., 2002; Bao et al., 2005; Kim et al., 2017; Li et al., 2007). In contrast, glycan arrays have the advantages of low dosage, high sensitivity, high throughput, and rapid analysis, which is suitable for the large-scale screening and investigation of potential biological functions of various glycans and their conjugates including CS/DS poly- and oligosaccharides (Fukui et al., 2002). By using this technique, E unit-rich polysaccharides and structure-defined tetrasaccharides have been shown to interact with TNF-α (Tully et al., 2006), bFGF (Miyachi et al., 2015), and midkine-derived and brain-derived neurotrophic factor (Gama et al., 2006) with high affinity. Moreover, this method was used to investigate the interaction between DS and its binding proteins (Yamaguchi et al., 2006). Thus, glycan arrays can be very a powerful tool for the identification of novel functions of CS/DS oligosaccharides with different structures derived from the digestion of CS/DS polysaccharides with various degrading enzymes.

**FIGURE 4:**
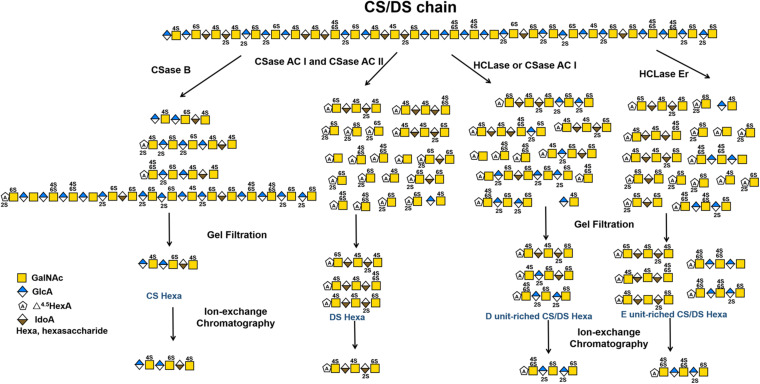
Schematic diagram of preparation of various structure-defined hexasaccharides by CS/DS-degrading enzymes with specific activity.

The selective degradation of inactive domains is an ideal way to isolate functional oligosaccharides from CS/DS polysaccharides. Technically, this can be achieved by selecting enzymes with a certain substrate specificity. For example, in a previous study we found that shark skin/liver-derived CS/DS could strongly interact with PTN and HGF to promote neurite outgrowth and this activity could be abolished by treatment with the CSase B but not the CSase AC I. Based on this finding, a PTN- and HGF-binding hexasaccharide containing two iB units was eventually isolated from shark skin CS/DS through selective digestion with the CSase AC I followed by pleiotrophin affinity and anion exchange chromatographies (Li et al., 2007, 2010). In addition to the different selectivity of the CSase AC and B toward uronic acid epimers, some enzymes show high sensitivity to specific sulfation patterns of CS/DS chains, which is important for the preparation of oligosaccharides with specific structures, such as E unit-rich oligosaccharides, which play a key role in neuronal cell adhesion and outgrowth (Ueoka et al., 2000; Mikami et al., 2009; Nandini and Sugahara, 2006; Purushothaman et al., 2007), cancer cell metastasis (Li et al., 2008; Mizumoto et al., 2012, 2013b) and virus infections (Bergefall et al., 2005; Kato et al., 2010), and D unit-rich oligosaccharides, which significantly promote hippocampal neurite outgrowth through interacting with various growth factors and other proteins (Nandini and Sugahara, 2006; Clement et al., 1998; Shida et al., 2019). Testicular hyaluronidase can efficiently digest non-sulfated and low sulfated domains but not highly sulfated domains in CS chains, which makes it a good choice for the selective isolation of highly sulfated domains from CS/DS chains, such as the preparation of D unit-rich oligosaccharides from CS-D (Nadanaka and Sugahara, 1997) and E unit-rich oligosaccharides from CS-E (Kinoshita et al., 2001), respectively. Furthermore, our studies have shown that HCLase like CSase AC I cannot cleave the β–1,4–linkage between GalNAc and D unit (Han et al., 2014), and in contrast, HCLase Er cannot efficiently act on the β–1,4–linkage between E unit and GlcA (Peng et al., 2018), which makes these enzymes more specific tools for the selective isolation of D unit-rich domains and E unit-rich domains in CS/DS chains. Figure 4 shows a schematic diagram of the preparation of various structurally determined hexasaccharides by CS/DS degradation of enzymes with specific activities. In addition, various CS/DS sulfatases combined with sulfotransferases can be potential tools to further edit the sulfation pattern of the obtained oligosaccharides for functional evaluation (Li et al., 2017; Shioiri et al., 2016; Wang et al., 2015; Wang et al., 2019b). Recently, Li et al. developed a method to synthesize CS oligosaccharides using multiple glycosyltransferases and sulfotransferases, and synthesized 15 homogeneous CS oligosaccharide by this method (Li et al., 2017). However, the de novo synthesis of CS/DS oligosaccharides is very cumbersome, heavy workload, time-consuming and costly. By contrast, it can be a relatively simple, efficient and low-cost choice to prepare basic oligosaccharide structures from CS/DS polysaccharides by treatment with various degrading enzymes and further modify these basic structures with specific synthetases to prepare structure-defined oligosaccharides (Cai et al., 2012; Zhang et al., 2019).

In brief, CS/DS-degrading enzymes, including hydrolases, lyases and sulfatases, are very useful tools for preparing functional oligosaccharides with specific structures from CS/DS polysaccharides. With the identification of an increasing number of enzymes with novel substrate specificity, the directional isolation and preparation of oligosaccharides with specific structures from various CS/DS forms will be achieved, which may represent a more feasible and low-cost way to prepare CS/DS oligosaccharides with specific bioactivity compared with synthetic methods.

### Sequencing of CS/DS Oligosaccharides

Sequencing of the isolated functional oligosaccharides with special structures is essential for structure-function studies of CS/DS. Various methods, such as NMR and MS, have been used to determine the structures of CS/DS oligosaccharides. Ly et al. reported successful sequencing of bikunin CS chains with up to 43 saccharide units with FT-ICR-MS/MS (Ly et al., 2011). It is still difficult to distinguish different units with the same sulfation degree, such as monosulfated A and C units or disulfated D and E units. Meanwhile, the distinction of hexuronate epimers and the loss of sulfate groups during analysis are also major challenges. In addition, instrument requirements formethods using NMR and MS are more sophisticated, and NMR, in particular, requires a much greater amount of sample. Sugahara lab has developed a method to analyze and monitor CS/DS oligosaccharides with highly sensitively via the fluorescent labelling of reducing ends with 2-AB followed by anion exchange HPLC, and the detection limit for 2-AB-oligosaccharides is as low as 1 pmol (Kinoshita and Sugahara, 1999). However, further study showed that the 2-AB labelling completely inhibited the cleavage of the β-1,4- linkage in the tetrasaccharide at the reducing end of the CS/DS chain by CSase ABC (Kinoshita and Sugahara, 1999). Whereas, in most cases, some exolyases, such as CSase AC II and HCDLase, can effectively degrade 2-AB-labelled CS oligosaccharides to release the 2-AB-labelled reducing-end disaccharides. By taking advantage of these characteristics of enzymes, CS/DS oligosaccharides that are shorter than decasaccharides can be easily sequenced by enzymatic methods (Bao et al., 2005; Deepa et al., 2007b). Recently, we exploited a novel exolyase HCDLase combined with CSase ABC to sequence a complex octasaccharide (ΔC-A-D-C) (Wang et al., 2017). In this method, the disaccharide composition of the octasaccharide was determined by digestion with the CSase ABC followed by 2-AB labelling and HPLC assay. Then, the octasaccharide was labeled with 2-AB and partially digested with CSase ABC to prepare the 2-AB-labelled reducing-end hexasaccharides. The reducing-end C unit can be directly determined through the digestion of the 2-AB-labelled octasaccharide with HCDLase followed by HPLC-fluorescence detection. To determine the first and second disaccharide units from the nonreducing terminus, the 2-AB-labelled octasaccharide and reducing-end hexasaccharide prepared as described above were individually digested by the CSase ABC and analyzed by HPLC after relabelling with 2-AB. This strategy is theoretically feasible for sequencing longer oligosaccharides but is not easy to achieve due to the rapid increase in operating steps with the increase of saccharide chains. Moreover, Shioiri et al. developed an enzymatic method for the sequencing of a synthesized CS dodecasaccharide (C-C-O-A-O-O) by using a strategy involving dual-fluorescence labelling and dual-digestion (Shioiri et al., 2016). This provides a possibility of sequencing longer CS/DS oligosaccharides. However, the inability of testicular hyaluronidase to cleave DS and highly sulfated CS limits the application of this method, and thus, it is necessary to find alternative enzymes with better features.

### Potential Medical Applications

The abnormal expression of CS/DS has been shown to be closely related to the occurrence and development of various diseases, such as glial scar formation after brain injury, tumor metastasis, skeletal disorder and viral infection, indicating that the treatment with CS/DS-degrading enzymes should affect the progression of the related diseases and these enzymes might be used as therapeutic agent for the related diseases. In the research of a spinal cord injury (SCI) model, Lemons et al. expounded that CSPGs increased in the lesion and inhibited the growth of axons, that is, inhibited the recovery of the function of the lesion (Lemons et al., 1999; Mondello et al., 2015). Injecting of CSase ABC induces abnormal axon growth or enhance axon regeneration in zebrafish (Bernhardt and Schachner, 2000; Becker and Becker, 2002), adult rats (Bradbury et al., 2002; Janzadeh et al., 2017; Moon et al., 2001), mice (Li et al., 2013), and cats (Mondello et al., 2015). Moreover, CSase ABC combined with other operations such as human adipose derived stem cells (Sarveazad et al., 2017) or low level laser therapy (Janzadeh et al., 2017) can promote the treatment of SCI. Additionally, CSase ABC is used to treat some diseases related to nerve damage, such as glaucoma (Tribble et al., 2018), lumber intervertebral disc (Hoogendoorn et al., 2007; Sugimura et al., 1996; Lü et al., 1997), and to delay the progress of Parkinson’s disease (Kauhausen et al., 2015) and Alzheimer’s disease (Howell et al., 2015). Overall, these studies suggest that CS/DS-degrading enzymes, in particular CSase ABC with broad substrate spectrum, are very promising therapeutic agents for the treatment of nerve injury-related diseases. Most recently, studies have also found that 4-*O*-sulfated CS GAG chains are increased significantly at the injury site after SCI (Wang et al., 2008) and optic nerve injury (Pearson et al., 2018). ARSB, a mammalian 4-*O*-sulfatase, treatment improves locomotor function recovery after SCI (Yoo et al., 2013) and improves regeneration after optic nerve injury (Pearson et al., 2018). Comparing with the exo-sulfatases, the newly discovered 4-*O*-endosulfatases may show better effect in nerve injury repair, which remains to be investigated.

The abnormal expression of CS/DS or CS/DSPGs in cells and tissues is closely related to many tumorigenic processes including cell growth and survival, adhesion, migaration and invasion (Theocharis et al., 2010; Iozzo and Sanderson, 2011; Li et al., 2008). A series of studies have shown that CSases have potential application value in anti-tumor. For example, CSase ABC and CSase AC could significantly inhibited the growth of tumor while streptomyces hyaluronidase, and β-glucuronidase could not (Takeuchi, 1972), CSase AC and CSase B can inhibits the invasion and proliferation of melanoma (Denholm et al., 2001), CSase ABC can assist temozolomide in the treatment of glioblastoma (Jaime-Ramirez et al., 2017), the adhere ability of squamous tongue carcinoma SCC-9 LN-1 cells can be reduced by treatment with CSaseABC (Kawahara et al., 2014), the metastasis of Lewis lung carcinoma LM66-H11 cells can be effectively inhibited by treatment with CSase ABC (Li et al., 2008), and so on (Sullivan et al., 2018). Moreover, Link et al. found that treatment with CSase ABC enhanced integration of both immature and mature self-assembled articular cartilage to native tissue, indicating that Case ABC has a potential therapeutic target for the integration of neocartilage (Link et al., 2020). In fact, some hyaluronidases, such as ovine testicles hyaluronidase (Vitrase^®^), bovine testicular hyaluronidase (Hydase^TM^) and recombinant human hyaluronidase PH20 (ENHANZE^®^), have been clinically used in ophthalmic surgery (Sarvela et al., 1994) and in cosmetic dermatosurgery for the treatment of complications caused by filler injection (Hirsch et al., 2007).

Additionally, the lack of sulfatase leads to the long-term accumulation of highly sulfated oligosaccharides in the lysosome, which cause lysosomal storage disorders MPSs. MPSII (Hunter, OMIM 309900), MPS IVA (Morquio A, OMIM 253000) and MPS VI (Maroteaux-Lamy, OMIM 253200) resulted from the deficiencies of IDS, GALNS and ARSB, respectively (Bondeson et al., 1995; Matalon et al., 1974; Dorfman et al., 1976). Enzyme replacement therapy is the standard treatment option for MPSs, which can start treatment immediately and improve prognosis. Recombinant human IDS (idursulfase and idursulfase beta), GALNS (elosulfase alfa) and ARSB (galsulfase) are clinically used to treat the corresponding MPSs (Whiteman and Kimura, 2017; Sawamoto et al., 2020; Harmatz and Shediac, 2017). Hematopoietic stem cell transplantation is also available for MPSs treatment (Coppa et al., 1995; Mullen et al., 2000). Moreover, gene therapy should be another potential choice for MPSs (Sawamoto et al., 2018; Ponder and Haskins, 2007).

However, the clinical application of CS/DS-degrading enzymes still faces many problems such as immunogenicity, instability and low activity in vivo, which need further study to solve.

## Conclusion

Undoubtedly, CS/DS-degrading enzymes with various specific activities play indispensable roles in structural and functional studies as well as other applications related to CS/DS, such as disaccharide composition analysis, quality control of products, preparation of bioactive oligosaccharides, and oligosaccharide sequencing. However, the types and numbers of well-characterized enzymes currently are far from meeting the needs of the research and applications of CS/DS. Therefore, it is an urgent task to identify more CS/DS-degrading enzymes with novel specific activity and to carry out re-examination of old enzymes whose action patterns remain to be investigated in detail.

## Author Contributions

WW, LS, and YQ collected the literature, wrote the manuscript, and made the figures. FL conceptualized, edited and made significant revisions to the manuscript. All authors read and approved the final manuscript.

## Conflict of Interest

The authors declare that the research was conducted in the absence of any commercial or financial relationships that could be construed as a potential conflict of interest.
